# Gamma Ray-induced Mutations in *pyrEF* Genes in *Frankia casuarinae* Strain CcI3

**DOI:** 10.1264/jsme2.ME24062

**Published:** 2025-03-12

**Authors:** Ken-ichi Kucho, On Han, Miki Yunoki

**Affiliations:** 1 Graduate School of Science and Engineering, Kagoshima University, 1–21–35 Korimoto, Kagoshima 890–0065, Japan; 2 Faculty of Science, Kagoshima University, 1–21–35 Korimoto, Kagoshima 890–0065, Japan

**Keywords:** 5-fluoroorotic acid, gamma rays, insertion sequences, uracil auxotrophs

## Abstract

*Frankia* spp. are multicellular actinobacteria with the ability to fix atmospheric dinitrogen (N_2_). *Frankia* fixes N_2_ not only in the free-living state, but also in root-nodule symbioses with more than 200 plant species called actinorhizal plants. In the present study, we isolated mutants of the *pyrE* (orotate phosphoribosyltransferase) and *pyrF* (orotidine-5′-phosphate decarboxylase) genes in *Frankia casuarinae* strain CcI3 using gamma rays as a mutagen and systematically identified the types of mutations that occurred in these genes. *pyrEF* mutants were isolated as uracil auxotrophs using the antimetabolite 5-fluoroorotic acid. We elucidated the nucleotide sequences of the *pyrEF* genes in 32 uracil auxotrophs, and detected eight substitutions, 17 single-nucleotide deletions, and seven large insertions. Large insertions were insertion sequences (IS elements); four belonged to the IS*4* family, two to the IS*66* family, and one to the IS*110* family. This is the first study to demonstrate the mobilization of IS elements in the *Frankia* genome.

The genus *Frankia* belongs to the class *Actinobacteria*, order *Frankiales*, and family *Frankiaceae*. *Frankia* species possess the unique ability to fix atmospheric dinitrogen (N_2_), a trait not observed in other actinobacterial genera. N_2_ fixation occurs within vesicles that form at the tips of hyphae. These vesicles are encapsulated by multiple layers of hopanoid lipids, serving as a barrier against oxygen (Berry *et al.*, 1993). Nitrogenase, an oxygen-sensitive enzyme, is exclusively synthesized within these vesicles (Meesters, 1987), enabling *Frankia* to effectively fix N_2_ even under aerobic conditions.

*Frankia* establishes root nodule symbioses with more than 200 plant species across eight families, collectively known as actinorhizal plants (Benson and Dawson, 2007). Within these nodules, *Frankia* performs N_2_ fixation and supplies the products to the host plants. This symbiotic relationship enables actinorhizal plants to grow rapidly even in nutrient-poor soils. Consequently, actinorhizal plants serve as vital pioneer species during the revegetation of disturbed lands. A phylogenetic ana­lysis classified *Frankia* strains into four lineages, each with distinct host preferences (Pozzi *et al.*, 2018; Gtari *et al.*, 2019). Lineage 1 comprises three subgroups (1a, 1b, and 1c), with 1a and 1b infecting plant species in Myricaceae and the genus *Alnus* of Betulaceae, while 1c infects Casuarinaceae. Lineage 2 infects plants from four families within the Rosales and Cucurbitales orders. Lineage 3 has a broader host range, infecting plant species from several families within the Fagales and Rosales orders. Lineage 4 comprises strains unable to fix N_2_ or reinfect host plants.

To genetically identify novel *Frankia* genes involved in N_2_ fixation, we isolated mutants that were unable to fix N_2_ using 1-methyl-3-nitro-1-nitrosoguanidine (NTG) and gamma rays (GR) as mutagens (Kucho *et al.*, 2017). Mutants exhibited various phenotypes related to vesicle development (Kucho *et al.*, 2017; Asukai and Kucho, 2020) and symbiosis (Kucho *et al.*, 2023). In the NTG-induced mutant N3H4, we isolated cell lineages that restored N_2_ fixation. We re-sequenced the genomes of the mutant and restored cell lineages using a short-read type next-generation sequencer and comprehensively identified nucleotide substitutions and short indels (Kucho *et al.*, 2023). A comparative ana­lysis of these mutations detected a suppressor mutation in the NAD^+^ synthetase gene (Francci3_3146) of the restored cell lineages, and proved that this gene was responsible for aberrant N_2_ fixation (Kucho *et al.*, 2023). The same strategy was applied to GR-mutagenized mutants and their restored cell lineages. However, neither suppressor mutations nor reversions were detected (K. Kucho, unpublished). This result suggests that GR irradiation induced long insertions and deletions that were not detectable by our short-read mapping ana­lysis.

In the present study, we investigated mutation types (*e.g.*, substitutions, short indels (a few bp), and long indels) induced by GR irradiation in *Frankia*. We previously established a method to selectively isolate mutants of the *pyrE* (orotate phosphoribosyltransferase) and *pyrF* (orotidine-5′-phosphate decarboxylase) genes in *Frankia* using the antimetabolite 5-fluoroorotic acid (5-FOA) with ethyl methanesulfonate as a mutagen (Kakoi *et al.*, 2014). Briefly, 5-FOA is converted to 5-fluorouridine monophosphate (5-FUMP) by successive reactions catalyzed by the PyrE and PyrF proteins. Since 5-FUMP is toxic, wild-type (WT) cells cannot survive in the presence of 5-FOA. Mutants of the *pyrE* and *pyrF* genes are resistant to 5-FOA, but cannot synthesize uridine monophosphate (UMP), which is essential for growth. However, the growth of mutants may be supported by supplementing the medium with uracil. Therefore, *pyrE* and *pyrF* mutants (uracil auxotrophs) may be selectively isolated on minimal media containing 5-FOA and uracil. We utilized this method as a tool to easily identify mutations induced in GR-irradiated *Frankia casuarinae* strain CcI3 cells, which is a symbiont of *Casuarina* and *Allocasuarina* plant species (Zhang *et al.*, 1984).

## Materials and Methods

### Bacterial strains and media

*F. casuarinae* strain CcI3 (Nouioui *et al.*, 2016) was used as the parental WT strain. Buffered ammonium propionate medium used for transformation (BAP-T) (Kucho *et al.*, 2009), a defined minimal medium, was employed to propagate WT *Frankia* cells.

### Isolation of uracil auxotrophs with 5-FOA

Uracil auxotrophic mutants carrying mutations in the *pyrE* and *pyrF* genes were selectively isolated using 5-FOA and uracil according to the procedure described by Kakoi *et al.* (2014). Briefly, *Frankia* CcI3 cells grown in BAP-T medium were collected from a 14-mL culture by centrifugation and resuspended in 3 ml BAP-T. Hyphae were homogenized by forced passages through a 21G needle (TERUMO) five times and irradiated with GR from ^60^Co (772 or 1,158 Gy). The two doses were tested because we used conditions for the isolation of N_2_ fixation mutants (Kucho *et al.*, 2017). Cells were cultivated in BAP-T supplemented with 100‍ ‍μg mL^–1^ uracil and 100‍ ‍μg mL^–1^ uridine at 28°C for 2 days. Cell suspensions were plated on solid CB media (Bassi and Benson, 2007) containing 0.5‍ ‍mg mL^–1^ 5-FOA, 100‍ ‍μg mL^–1^ uracil, and 100‍ ‍μg mL^–1^ uridine, and were incubated for 2 months.

### Confirmation of uracil auxotrophy

Colonies that appeared on media containing 5-FOA were propagated in 0.5‍ ‍mL BAP-T with 100‍ ‍μg mL^–1^ uracil. Cells were washed twice with sterilized distilled water to remove uracil, and half of them were inoculated into BAP-T and the remaining half into BAP-T with 100‍ ‍μg mL^–1^ uracil. They were incubated at 28°C for one month, and cell density was assessed as OD_620_. Strains that grew in BAP-T with uracil, but not in BAP-T without uracil were considered to be uracil auxotrophic mutants.

### Identification of mutations in *pyrEF* genes

DNA segments containing the *pyrE* and *pyrF* genes were amplified by PCR using cell suspensions of the mutants as templates. Tks Gflex DNA polymerase (Takara) was used according to the manufacturer’s instructions. The primers 5′-GATGACATGAGGCAACGGT-3′ and 5′-ATCATGCTTCTACACGGGCT-3′ were used for *pyrE*, and 5′-ATCGTTGCAGGAATACCACG-3′ and 5′-AAGGCTTTGACGGACGAAAG-3′ for *pyrF*. Amplified products were electrophoresed on a 0.8% agarose gel containing 1×TAE buffer (40‍ ‍mM tris-acetate and 2‍ ‍mM EDTA, pH 8.0). To identify mutations in the amplified products, Sanger sequencing was performed by Eurofins Genomics. Detailed similarity searches for IS sequences were conducted using the ISfinder database (https://www-is.biotoul.fr/blast.php).

## Results

### Isolation of uracil auxotrophic mutants

**Fig. 1.**
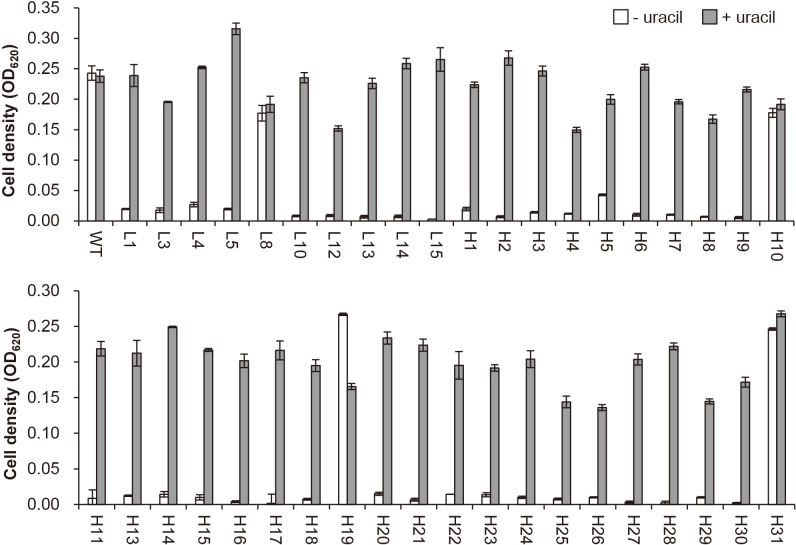
Confirmation of uracil auxotrophy. Wild-type (WT) and 5-FOA-resistant strains were cultivated in minimal media with (gray box) or without (open box) uracil for one month. Strains obtained from 772-Gy (L1 to L15) and 1,158-Gy (H1 to H31) GR irradiation were tested. The average, along with the standard deviation, of three technical replicates is shown.

Hyphae of *Frankia* CcI3 were irradiated with GR (772 and 1,158 Gy), and 17 and 85 5-FOA-resistant (5-FOA^R^) colonies were obtained, respectively. The rate of 5-FOA^R^ colonies ranged between approximately 2×10^–3^ and 4×10^–3^. Forty 5-FOA^R^ colonies were isolated. They were cultivated in minimal media with or without uracil to test uracil auxotrophy (Fig. 1). While WT cells grew regardless of the presence of uracil, 36 of the 40 5-FOA^R^ strains only grew in the uracil-containing medium, indicating that they were uracil auxotrophic mutants. Mutant strains L1, L3 to L5, L10, and L12 to L15 were derived from 772-Gy GR irradiation. Mutant strains H1 to H9, H11, H13 to H18, and H20 to H30 were derived from 1,158-Gy GR irradiation. The remaining four strains (L8, H10, H19, and H31) also grew in the uracil-deficient medium, suggesting that they were false positives from screening.

### Identification of mutations in *pyrEF* genes

**Fig. 2.**
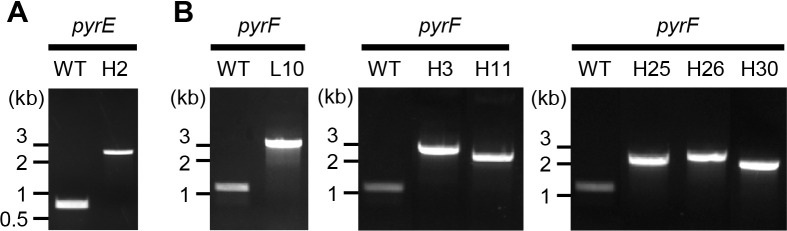
PCR amplification of *pyrEF* genes in insertional mutants. Gel electrophoresis was performed on the PCR-amplified *pyrE* (A) and *pyrF* (B) genes. In the wild-type (WT) strain, the expected sizes of the amplified products were 0.73‍ ‍kbp for *pyrE* and 1.1‍ ‍kb for *pyrF*, respectively.

To identify mutations in the *pyrEF* genes, both genes were amplified by PCR from the uracil auxotrophs. Electrophoresis of the amplified products detected bands with larger sizes than WT in *pyrE* of strain H2 and *pyrF* of strains L10, H3, H11, H25, H26, and H30 (Fig. 2), which was indicative of the insertion of large DNA fragments within the genes. The other mutant strains showed bands of the same size as WT (data not shown).

The nucleotide sequences of the amplified products were elucidated to identify mutations carried by 32 uracil auxotrophs. All the mutations detected are listed in Table S1. In summary, 10 mutations in *pyrE* and 22 mutations in *pyrF* were detected. The numbers of mutations roughly corresponded to the lengths of the genes (*pyrE* 537 bp and *pyrF* 825 bp). One-quarter of the mutations were nucleotide substitutions, consisting of seven transitions and one transversion (Table 1). More than half (17) of the mutations were single-nucleotide deletions that caused a frameshift in translation. Sixteen of the 17 deletions occurred at the 388th G in *pyrF*.

**Fig. 3.**
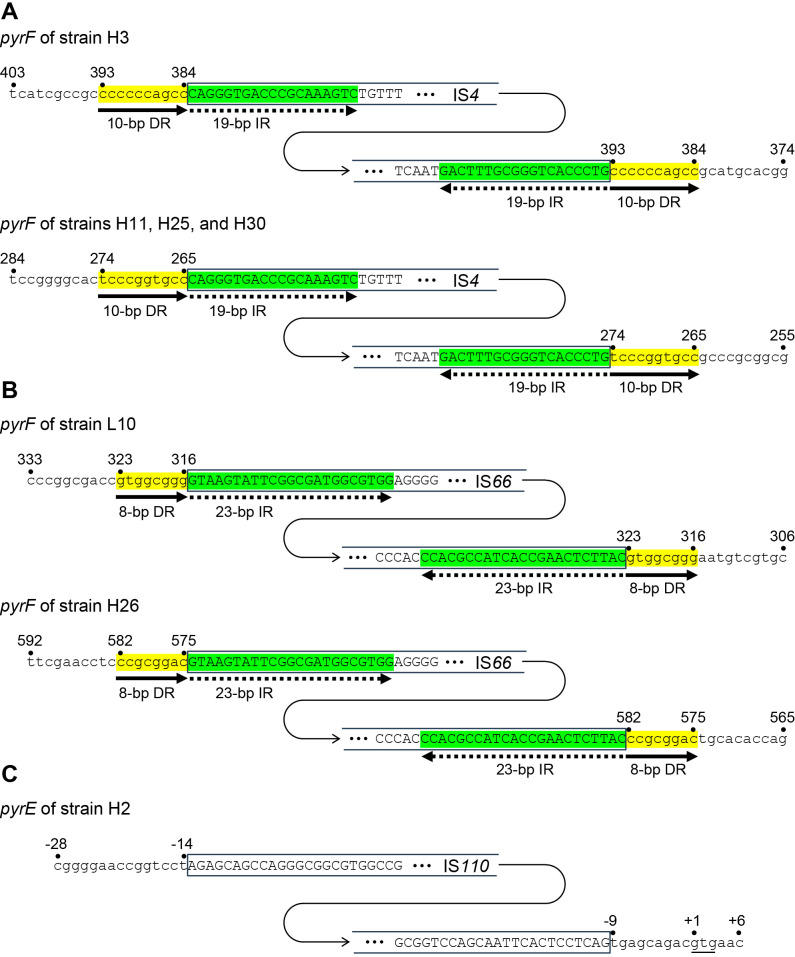
Nucleotide sequences of insertion sites of IS*4* (A), IS*66* (B), and IS*110* (C) elements. Lowercase letters represent the nucleotide sequences of the *pyrE* and *pyrF* genes, and uppercase letters represent those of the IS elements. Direct repeats (DR) are indicated by solid arrows and yellow highlights. Inverted repeats (IR) are indicated by dashed arrows and green highlights. Numbers represent positions relative to the translation initiation codon. An initiation codon (gtg) of the *pyrE* gene is underlined (C).

Seven mutations were large insertions as described above. Nucleotide sequencing revealed that these large insertions were insertion sequences (IS), a type of mobile genetic element (Fig. 3). Six mutants had IS in the coding region of *pyrF*. Four of the *pyrF* mutants (H3, H11, H25, and H30) had IS*4* elements with an identical sequence (Fig. 3A). The sequences of the IS*4* elements were identical to those of the Francci3_2330 and Francci3_2941 genes (the two genes have identical sequences). The remaining two *pyrF* mutants (L10 and H26) had IS*66* elements with an identical sequence (Fig. 3B), and the sequences of the IS*66* elements were identical to those of the Francci3_0277 and Francci3_1922 genes (the two genes have identical sequences). One of the seven insertion mutants (H2) had an IS*110* element in the promoter region of *pyrE* (Fig. 3C). The sequence of the IS*110* element was identical to the Francci3_3436 gene.

At the IS*4* insertion sites, the partial sequences of the *pyrF* gene were duplicated, generating a 10-bp direct repeat (DR) (Fig. 3A). In addition, a 19-bp inverted repeat (IR) was found at both ends of the IS*4* elements. DR and IR were also found at the IS*66* insertion sites; DRs were 8 bp long and IRs were 23 bp long (Fig. 3B). Apparent DR and IR were not found at the IS*110* insertion site (Fig. 3C).

## Discussion

We previously isolated *F. casuarinae* mutants defective in nitrogen fixation using GR as a mutagen (Kucho *et al.*, 2017). Using a next-generation sequencer, we re-sequenced the genomes of the mutants and identified nucleotide substitutions and short indels spanning several base pairs (Kucho *et al.*, 2017). However, the short-read mapping ana­lysis conducted in that study overlooked large deletions and insertions. In the present study, we investigated mutation types (*e.g.*, substitutions and short and long indels) induced by GR‍ ‍in the *pyrEF* mutants isolated by positive selection screening using the antimetabolite 5-FOA.

The most frequently observed mutations were single-nucleotide deletions (17 of 32 mutants, Table 1). Sixteen of these 17 mutations occurred at the same site in *pyrF* (G388_del, Table S1). The deletion of this base in the *pyrF* gene was also frequently found in mutants induced by ethyl methanesulfonate (Kakoi *et al.*, 2014). Therefore, this single-nucleotide deletion did not appear to be a mutation specific to GR irradiation. The 388th G is one of seven consecutive Gs. In mono-nucleotide repeats, deletions frequently occur due to “replication slippage” (Viguera *et al.*, 2001). During replication slippage, the newly synthesized strand by DNA polymerase and the template strand temporarily dissociate and reattach in a misaligned manner. This misalignment leads to indel mutations.

Eight of the 32 mutants carried nucleotide substitutions (Table 1). The induction of nucleotide substitutions by GR has been reported in various organisms (Glickman *et al.*, 1980; Li *et al.*, 2019; Yoshihara *et al.*, 2013; Manaka *et al.*, 2024). Ionizing radiation, such as GR, is known to induce the formation of hydroxyl radicals through the radiolysis of H_2_O (Bjelland and Seeberg, 2003). These radicals may lead to the modification of DNA bases, resulting in substitutions.

We previously resequenced the genomes of GR-induced nitrogen fixation mutants (Kucho *et al.*, 2017). In these genome ana­lyses, deletions were not observed as frequently as in the present study of *pyrEF* genes. Nucleotide substitutions, *i.e.*, transitions (78% of all mutations) and transversions (15% of all mutations), were more common. Single-nucleotide deletions that were frequently observed in the *pyrF* gene (Table 1) occurred specifically in the consecu­tive‍ ‍G stretch, which is a hotspot for deletions by repli­cation‍ ‍slippage (Viguera *et al.*, 2001). However, these mono-nucleotide repeats were not common across the entire‍ ‍genome. As a result, the relative number of single-nucleotide deletions to nucleotide substitutions (*i.e.*, transitions and transversions) was lower than in the *pyrF* gene.

Seven of the 32 mutants had IS elements in the *pyrEF* genes (Table 1 and Fig. 3). ISs are the smallest mobile genetic elements, encoding only the enzymes necessary for their transposition, and are capable of repeated insertion into many different sites within a genome. IS elements are classified into major families based on the amino acid sequence of the transposase, terminal IR sequences, and flanking DR sequences generated upon insertion (Siguier *et al.*, 2014; Siguier *et al.*, 2015). Several environmental factors are known to induce the transposition of ISs in bacteria, such as UV irradiation, microaerobic conditions, oxidative stress, metals, antibiotics, and high temperatures (Vandecraen *et al.*, 2017). In the radiation-resistant bacteria *Deinococcus radiodurans* and *D. geothermalis*, irradiation by GR induced the transposition of ISs. (Mennecier *et al.*, 2006; Ye *et al.*, 2022). In the present study, mutagenesis was performed using two doses of GR (772 and 1,158 Gy), and more IS transpositions were observed at the higher dose. Therefore, the transposition of these *Frankia* ISs may be activated by GR irradiation. The genome of *F. casuarinae* strain CcI3 was previously shown to be very rich in ISs (Normand *et al.*, 2007), containing 148 ISs belonging to 12 families, which account for 3% of all coding sequences (Bickhart *et al.*, 2009).

In the present study, the most numerous transposed IS element was IS*4* (4 out of 7). IS*4* is the most dominant IS family in *F. casuarinae* CcI3, with more than 50 homologs present in the genome (Bickhart *et al.*, 2009). Sequence ana­lyses revealed that transposed IS*4* homologs in the four mutants were the Francci3_2330 and Francci3_2941 genes. Since three of the four mutants had IS*4* insertions at the same locus (Fig. 3A), this IS element may prefer a specific targeting site. More detailed similarity searches revealed that the Francci3_2330/2941 genes belong to the IS*As1* family. IS*As1* was identified in more than 50 bacterial species, including the fish pathogen *Aeromonas salmonicida* (Ross *et al.*, 2021). IS*As1* contains a 14- to 22-bp terminal IR starting with “CAGGG”, and a similar IR was found in the Francci3_2330/2941 genes (Fig. 3A). Additionally, a 10-bp DR identified at the insertion site in other bacteria was present in our *Frankia* mutants (Fig. 3A).

The second most numerous transposed IS element was IS*66* (2 out of 7). A previous study identified 16 IS*66* homologs in the strain CcI3 genome (Bickhart *et al.*, 2009). The transposed IS*66* homologs in these two mutants were the Francci3_0277 or Francci3_1922 genes. IS*66* was initially discovered in the Ti plasmid of *Agrobacterium tumefaciens* (Machida *et al.*, 1984) and shortly after in a symbiotic plasmid of *Rhizobium fredii* (Ramakrishnan *et al.*, 1986). Most IS*66* members are found in Proteobacteria, with some also originating from Bacteroidetes/Chlorobi and Firmicutes. IS*66* contains a 20- to 30-bp terminal IR starting with “GTAAGCG” (Ross *et al.*, 2021), and similar IR was found in the Francci3_0227/1922 genes (Fig. 3B). Additionally, 8-bp DRs characteristic of the insertion site were present in our *Frankia* mutants (Fig. 3B).

The transposition of IS*110* was detected in mutant strain H2. Nine IS*110* homologs were previously shown to be pre­sent in the strain CcI3 genome (Bickhart *et al.*, 2009). IS*110* was initially discovered in the actinomycete *Streptomyces coelicolor* and has since been found in nearly 130 bacterial and archaeal species (Ross *et al.*, 2021). The‍ ‍transposed IS*110* homolog in the mutants was Francci3_3436. IS*110* is notable in that it lacked DR and IR, and these sequences were also not found in our case (Fig. 3C).

This is the first study to demonstrate the mobilization of IS elements in the *Frankia* genome. Some N_2_ fixation mutants (Kucho *et al.*, 2017) may have been caused by insertional mutations by these IS elements. This result is expected to provide novel insights into the diversity and evolution of *Frankia* genomes. Additionally, the use of these IS elements as mutagens will be useful for genetic studies on the biology of *Frankia*.

## Citation

Kucho, K., Han, O., and Yunoki, M. (2025) Gamma Ray-induced Mutations in *pyrEF* Genes in *Frankia casuarinae* Strain CcI3. *Microbes Environ ***40**: ME24062.

https://doi.org/10.1264/jsme2.ME24062

## Supplementary Material

Supplementary Material

## Figures and Tables

**Table 1.** Summary of mutations detected in *pyrEF* genes.

Type of mutation	Number
Substitution (transition)	7
Substitution (transversion)	1
Frame shift (single-nucleotide deletion)	17
Large insertion	7
Total	32
