# Supplementary material for: Gamma Ray-induced Mutations in pyrEF Genes in Frankia casuarinae Strain CcI3
Source: Microbes Environ. 2025 Mar 12;40(1):ME24062. doi: 10.1264/jsme2.ME24062 (PMC11946415; doi:10.1264/jsme2.ME24062)
Supplement: Supplementary file 1 — Supplementary Material [file 40_24062_s1.pdf]

Table S1. Mutations identified in each uracil auxotroph strains.

Strain[a]	Gene	Mutation
L1	*pyrE*	G311A[b]
L3	*pyrF*	G388_del[c]
L4	*pyrE*	G194A
L5	*pyrF*	G388_del
L10	*pyrF*	Large insertion (IS*66*)
L13	*pyrE*	G311A
L14	*pyrF*	G388_del
H1	*pyrF*	G388_del
H2	*pyrE*	Large insertion (IS*110*)
H3	*pyrF*	Large insertion (IS*4*)
H4	*pyrE*	C442T
H5	*pyrE*	T212G
H6	*pyrF*	G388_del
H7	*pyrF*	G388_del
H9	*pyrE*	G404_del
H11	*pyrF*	Large insertion (IS*4*)
H13	*pyrE*	G194A
H14	*pyrF*	G388_del
H15	*pyrF*	G388_del
H16	*pyrF*	G388_del
H17	*pyrF*	G388_del
H18	*pyrF*	G388_del
H20	*pyrF*	G388_del
H21	*pyrF*	G388_del
H22	*pyrF*	G388_del
H23	*pyrE*	G311A
H24	*pyrF*	G388_del
H25	*pyrF*	Large insertion (IS*4*)
H26	*pyrF*	Large insertion (IS*66*)
H28	*pyrF*	G388_del
H29	*pyrE*	G310A
H30	*pyrF*	Large insertion (IS*4*)

[a]Strains L1 to L14 were derived from 772-Gy GR irradiation and strains H11 to H30 were derived from 1158-Gy GR irradiation.

[b]The 311th nucleotide G in the WT was mutated to A in the mutant. The nucleotide position is relative to the first nucleotide of the initiation codon.

[c]The 388th nucleotide G in the WT was deleted in the mutant.
